# Supplementary material for: Using a 24 h Activity Recall (STAR-24) to Describe Activity in Adolescent Boys in New Zealand: Comparisons between a Sample Collected before, and a Sample Collected during the COVID-19 Lockdown
Source: Int J Environ Res Public Health. 2021 Jul 29;18(15):8035. doi: 10.3390/ijerph18158035 (PMC8345423; doi:10.3390/ijerph18158035)
Supplement: Supplementary file 1 [file ijerph-18-08035-s001.zip › ijerph-1287121-supplementary.pdf]

Supplementary Table S1: Coding of possible activity reported in STAR-24

Number	Activity
1	Sleeping
2	Activities of daily living<br>• Showering, Bathing<br>• Getting dressed, getting ready or school, pottering around<br>• Meal preparation<br>• House work (cleaning, vacuuming, doing laundry etc)
3	Driving a car or riding in a car/bus/train/escooter
4	Active Travel<br>• Travelling by bike<br>• Walking to school/work etc<br>• Skateboarding/scootering for travel purposes
5	At school
6	Active PE class
7	Work/job<br>Home work
8	General every day activities<br>• Including hanging out with friends/family<br>• Playing/practicing musical instrument<br>• Listening to music<br>• Reading
9	Watching TV/Movies/streaming video content<br>General internet use (google/social media/chat/other)
10	Gaming<br>• PC<br>• Playstation<br>• Xbox<br>• Switch<br>• Mobile etc
11	Physical Activity (not organised sport)<br>• Walking/running for exercise<br>• Dancing/aerobics<br>• Swimming<br>• Riding a bike<br>• Resistance exercise (going to the gym, lifting weights, cross fit, F45, tribe etc)<br>• Skateboard/scooter<br>• Pilates/yoga<br>• Playing active game/throwing the ball around/mucking around but being active
12	Play or training for organised sport (rugby/football/basketball/netball/cricket/golf etc)<br>• Includes competitive gymnastics, track and field etc
13	Other (please specify)

Supplementary Table S2: Coding of possible intensities reported in STAR-24

Number	Intensity
1	Very light intensity (activities that involved little or no movement, breathing rate is slow)
2	Light intensity (activities that involve some movement, but do not elevate breathing rate)
3	Medium intensity activity (moving quickly/briskly, breathing rate is increased but you can still talk)
4	Hard/vigorous intensity activity (moving very quickly, breathing so hard you can't talk at the same time)

Supplementary Table S3. Median (25th, 75th) percentiles of 24-hour time-use (n=109)

	Full sample	Those who did the behaviour	
	Median (25th, 75th percentiles) minutes	n	Median (25th, 75th percentiles) minutes
Activities[a]			
Sleep	555 (510, 600)	109	555 (510, 600)
General	225 (180, 300)	109	225 (180, 300)
Transport	0 (0, 45)	54	45 (30, 90)
School/work	180 (105, 330)	92	195 (150, 338)
Activity	75 (45, 135)	99	75 (45, 135)
Screens	135 (75, 255)	98	150 (90, 270)
Gaming	30 (0, 120)	60	90 (60, 225)
Screens[b]			
No screen	405 (285, 540)	109	405 (285, 540)
Phone	90 (45, 210)	100	113 (45, 233)
TV	30 (0, 105)	65	90 (45, 165)
Computer	210 (45, 405)	94	240 (120, 435)
Tablet	0 (0, 0)	4	150 (83, 270)
Intensity[c]			
Very light	645 (510, 780)	104	660 (525, 780)
Light	135 (60, 285)	94	165 (75, 330)
Medium	30 (0, 60)	76	45 (30, 90)
Hard/vigorous	0 (0, 30)	50	30 (15, 60)
Posture			
Lying/reclining	615 (555, 675)	109	615 (555, 675)
Sitting	600 (495, 705)	109	600 (495, 705)
Standing	45 (30, 90)	94	60 (30, 90)
Stepping/moving	120 (60, 210)	104	128 (68, 218)

[a] ‘General’ included activities of daily living; ‘Transport’ included car/bus/train/e-scooter; ‘Activity’ included active transport, PE, sport, or other physical activity; ‘Screens’ included TV, computers, phones, not including gaming; and ‘Gaming’ included games played on PC, playstation, Xbox, Switch, mobile etc.

[b] If multiple screens in use then the one they were interacting with most was prioritised.

[c] Very light intensity described as: activities that involve very little or no movement, breathing rate is slow; Light intensity described as: activities that involved some movement, but do not elevate breathing rate; Medium intensity described as: moving quickly/briskly, breathing rate is increased but you can still talk;Hard/vigorous intensity described as: moving very quickly, breathing so hard you can’t talk at the same time.
